# A qualitative assessment of gender roles in child nutrition in Central Malawi

**DOI:** 10.1186/s12889-022-13749-x

**Published:** 2022-07-20

**Authors:** Elizabeth Mkandawire, Clement Bisai, Elizabeth Dyke, Anne Dressel, Hazel Kantayeni, Billy Molosoni, Peninnah M. Kako, Kaboni W. Gondwe, Lucy Mkandawire-Valhmu

**Affiliations:** 1 University of Pretoria, Old College House, University of Pretoria, Pretoria, South Africa; 2 CARE Malawi, Pamodzi House, Off Presidential Drive, Lilongwe, Malawi; 3 Gatineau, QC Canada; 4 University of Wisconsin-Milwaukee, College of Nursing, Cunningham Hall, Milwaukee, WI 53201 USA

**Keywords:** Child nutrition, Food Security, Inequalities, Care-giving, Low-income countries, Focus group

## Abstract

**Background:**

Child malnutrition persists globally with men and women playing distinct roles to support children’s nutrition. Women frequently carry the bulk of the workload related to food, care, and health, all of which are critical factors in child nutrition. For this reason, development efforts have emphasised women ignoring the potential role of men in supporting children’s nutrition. This study sought to understand the different roles that Malawian men and women play in children’s nutrition.

**Methods:**

This qualitative was conducted in rural Central Malawi as part of a baseline study in 2017 for the CARE Southern Africa Nutrition Initiative. Seventy-six participants were interviewed, including 19 men and 57 women, using focus group discussions and in-depth interviews. We sought to understand the gender distribution of men’s and women’s roles and how these roles influence child nutrition.

**Results:**

We found that both men and women were involved in productive, reproductive, and community work. However, consistent with the literature, women carried a disproportionate workload in supporting child nutrition compared to men. Women’s heavier workloads often prevented them from being able to meet children’s food needs. Nevertheless, shifts in gender roles were observed in some of the sampled communities, with men taking up responsibilities that have been typically associated with women. These changes in gender roles, however, did not necessarily increase women’s power within the household.

**Conclusions:**

Traditional gender roles remain prevalent in the sampled communities. Women continue to be primarily responsible for the food, care, and health of the household. Women’s heavy workloads prevent them from providing optimal care and nutrition for children. While efforts to advance gender equality by encouraging men to participate in child care and other household responsibilities appear to have had marginal success, the extent to which these efforts have successfully encouraged men to share power remains unclear. Improving gender equality and child nutrition will require efforts to redistribute gendered work and encourage men to move towards shared power with women over household decision-making and control over income.

## Background

Child malnutrition persists globally, even though child stunting has decreased by 10% over the past 6 years [1]. Malnutrition refers to a deficiency of nutrients and can include overnutrition, undernutrition and micronutrient deficiency [2]. While the three forms of malnutrition coexist in many African countries, undernutrition – a condition that results from inadequate intake of food that provides energy or nutrients – remains the most prevalent [3]. The rate of progress in addressing malnutrition globally is not sufficient to meet Sustainable Development Goal 2 targets on reducing stunting by 40% by 2025 [1]. Children’s nutrition is a fundamental human right, but it also has implications for economic growth [4]. Child malnutrition has significant implications for children’s capacity to meet their future potential [5]. In Africa, child malnutrition is estimated to result in losses of 1.6 to 16% in gross domestic product. Undernutrition is also attributed as a cause of death for 11% of African children aged 5 years and under [6].

In many African countries, child care – including child nutrition – falls under the purview of women. Women frequently carry the bulk of the workload related to food, care, and health, all of which are critical factors in child nutrition. Studies [7–9] suggest that women spend more of their income on children’s health, education and nutrition. When we look at tradition gender roles, women carry the primary responsibility for overcoming many of the underlying causes of malnutrition. Using focus group discussions and in-depth interviews conducted in four rural communities in Central Malawi, this study aimed to understand the roles that men and women play in child nutrition. While many studies indicate that women play a critical role in children’s nutrition, the use of the UNICEF framework [10] as a lens for analysis can assist us in determining the extent to which women can independently contribute to improved child nutrition outcomes given the multiple roles that they play.

The UNICEF conceptual framework, particularly the underlying causes of malnutrition, framed our analysis. We focussed in particular on access to food, adequate care for women and children and a healthy environment. The UNICEF framework defines food security as “...when all people at all times have physical and economic access to sufficient, safe^1^ and^2^ nutritious food to meet their dietary needs and food preferences for an active and healthy life” (3): 5 [11]). Care is defined as adequate care for women and children, including feeding practices. Health, in relation to the UNICEF framework specifically, is defined as access to adequate healthcare services and a healthy household environment [10].

### Gender roles and child nutrition

Traditional gender norms inform the diverse roles that men and women are expected to play to ensure household food security. These roles are typically informed by gender norms. Gender norms are behaviours, roles, and responsibilities that are socially allocated to men and women based on their sex. Gender norms also inform how men and women interact with one another [12].

Globally, women contribute to children’s nutrition in two ways. The first is through productive work, which includes farming and generating income to purchase food. These activities directly contribute to household food security. The second is through reproductive work, which is often unpaid and consequently considered less valuable [13]. Reproductive work entails multiple responsibilities which support household food security, including cooking, processing food, drawing water, cleaning, and collecting firewood [14]. These activities are undervalued because while they typically support child nutrition, they are not considered direct food production activities [15, 16].

Historically, women’s productive and reproductive work has been heavier than men’s [16–18]. Childcare has been a responsibility taken up by wives, older children, and female relatives. Time-use studies frequently underestimate women’s time spent on care activities because they rarely account for simultaneous tasks taking place. In many studies, women do not mention childcare as one of their duties unless prompted [19].

Access to clean water has implications for children’s nutrition, health and sanitation, and ultimately, the health of the household environment. The collection of water is highly feminised across Sub-Saharan Africa [20]. Water is used for domestic and economic purposes, including cleaning, cooking, caring for children, and tending to small-scale agriculture [21]. With only 50% of households in Sub-Saharan Africa having access to improved potable or proximal water sources [22], collecting water significantly burdens women and children who bear the primary responsibility for drawing water. As women and adolescent girls go back and forth collecting water, they spend a significant amount of time on this activity [23]. For example, 40% of rural households in Malawi do not have water on the premises and walk at least 30 minutes to a water source [24]. While collecting water is a necessity, the time spent on this activity has significant opportunity costs for women [25]. Drawing water adds to the burden of women’s unpaid domestic responsibilities, decreasing the time they could spend on income-generating and other non-essential activities such as leisure [26].

Men’s involvement has been considered a primary tool for transforming gender norms and working towards equality in terms of unpaid care work. Efforts to promote men’s shared responsibility for the family are driven by the need to redistribute reproductive^3^ work equally between men and women [27]. Target 5.4 of Sustainable Development Goal (SDG) 5 emphasises promoting shared family responsibility [28]. Evidence suggests that gender roles, particularly childcare and nutrition, are being challenged in Malawi, with men beginning to participate in these activities [29, 30]. However, the extent to which men actively participate in childcare and nutrition has not been widely investigated.

Many scholars [15, 19, 31] highlight the multiple roles women play within the household and broader community. These roles significantly influence their capacity to provide optimal care for children. Women’s disproportionate burden of productive and reproductive work appears to have remained the same over time in Malawi. In 2016, women’s time spent on unpaid care work was six times higher than men’s [32]. There is a paucity of literature highlighting men and women’s roles in food, care, and health and the impact these roles have on child nutrition. The limited literature generally focuses on one of the three underlying causes of malnutrition; i.e., inadequate access to food, inadequate care for women and children, insufficient health services and unhealthy environment without exploring how gender roles in these three areas combined could affect child nutrition [13, 14, 18, 29, 33, 34]. Policies and programmes that target women for nutrition interventions also increase women’s work burden [35]. Rao et al. [15] argue that it is crucial to understand how gendered time use affects nutrition outcomes.

This paper investigates the distribution of gendered roles, focussing in particular on exploring the extent to which women’s role in accessing food, providing adequate care for children and providing a health household environment can contribute to improved child nutrition outcomes. It also considers the implications of programmes aimed at redistributing women’s carework on gender equality.

## Methods

### Study design and sites

This study was a cross sectional descriptive study which was conducted as part of a baseline study conducted in 2017 for the CARE Southern Africa Nutrition Initiative (SANI). The SANI project was undertaken with the financial support of the Government of Canada through Global Affairs Canada. It aims to improve the persistent negative nutrition outcomes for women and children under 5 years of age in the Southern African countries of Malawi, Mozambique, and Zambia.

Between 2010 and 2015, Malawi made significant progress in reducing child malnutrition from 47 to 37% respectively [24, 36]. Nonetheless, child malnutrition, especially undernutrition, remains a significant challenge, with only 8% of children meeting the minimum acceptable diet (MAD). MAD refers to the number of children accessing food from at least four food groups and also measures the minimum meal frequency. Limited dietary diversity means that the majority of Malawian children are not accessing diverse foods to meet their daily dietary requirements. The 2021 Global Nutrition Report indicates that while Malawi is on track to meet the global targets for maintaining a low rate of overweight and wasting in children under five, it is not projected to meet other child nutrition targets including undernutrition and anemia [3].

The study took place in the Dowa and Ntchisi districts of rural Central Malawi. Four traditional authorities were sampled, including Dzoole, Kalumo, Kasakula, and Kayembe. Children’s nutrition in these districts was equal to or worse than the national average. Table 1 provides data from two districts on stunting, wasting, undernutrition, overweight anaemia and MAD in children aged five and under.

**Table 1:** Child nutrition in Dowa and Ntchisi

Town	Stunting	Wasting	Undernutrition	Overweight	Anaemia in children	MAD
Dowa	37%	2%	9%	5%	57%	10%
Ntchisi	40%	2%	11%	2%	56%	7%

Source: NSO and Macro, 2017

### Participants and sampling method

Data were collected from four different sub-samples of participants from one family unit. The sub-samples included 1) childbearing women with at least one child under 5 years of age; 2) men currently living with a female partner who also had at least one child under 5 years of age; 3) mothers-in-law of the women participants interviewed; and 4) community/religious leaders. The first three sub-samples were interviewed through focus group interviews, while the community/religious leaders were interviewed through in-depth individual interviews.

Seventy-six participants were sampled, including 19 men and 57 women. As the study was focused on maternal child nutrition, the emphasis was on women and under-five children. Data were collected from men and mothers-in-law to provide context. Mothers-in-law and also female community leaders constituted women in the study and participated in the interviews. The men were the husbands of the women who were sampled. All participants were above 18 years of age. A total of twelve focus group discussions (FGDs) were conducted. Three FGDs were conducted for each participant group (i.e. men, women, and mothers-in-law). We determined saturation when we noted that the themes were repetitive across the data. Four individual interviews were conducted with traditional leaders in each traditional authority.

All participants were recruited by CARE staff who visited each research site several days before data collection. Study participants were conveniently sampled directly from the community, with assistance from community leaders. Participants were conveniently sampled because this was a study conducted specifically for the purposes of informing the SANI project. As such, it was important to sample communities in which the SANI intervention would be implemented. Community leaders were included as study participants because it was culturally relevant and appropriate. It was respectful to seek their input in a study that had implications for their community. There was no indication that the data we garnered from community leaders were different from data gathered from other members of the community - the themes were confirmed across all the populations sampled for the study. Because the study areas were a distance from the main trading centre in all of the sampled communities, it was imperative that we identify people in the community who were available at the time that the study team was in the community. We confirmed themes across all populations and obtained data that were sensitive in nature, suggesting that participants were open about their experiences.

Two interviews were conducted closer to the trading centre, and two were further away from the trading centre. The selection of these two sites was based on the assumption that behaviours and perhaps even knowledge and potentially community cohesiveness would be different based on proximity to the trading centre. All interviews with the participants took place in a private space identified by the community, including churches, schools, or quiet spaces in the community.

Before each interview, written informed consent was obtained from all participants after verbally explaining the purpose of the study. The majority of the participants provided a thumbprint as evidence of consent to participate in the study because of limited formal education. Participants were assured of confidentiality and anonymity. They were also informed of the right to withdraw from the study at any time without penalty. During the focus group interviews, pseudonyms were used in the form of numbers to maintain participant anonymity. Participants thus addressed each other by number as opposed to by name throughout the focus group interviews.

### Data collection

The research team consisted of a qualitative research expert who trained three research assistants/facilitators to collect the qualitative data. Two male research assistants conducted FGDs with men. The two alternated facilitating and taking notes during the focus group interviews. The FGDs with women and the mothers-in-law were conducted by one of the female co-authors.

Data were collected over several weeks in July and August 2017. FGDs included four to eight participants in each focus group interview. Interviews lasted approximately one to 2 h and were recorded using a digital recorder. The interviews were transcribed first into Chichewa and then translated into English by the bilingual research assistants.

### Data analysis

The data were uploaded into Atlas.ti software. Data were iteratively coded and analysed using inductive qualitative analysis techniques. The first author reviewed all of the data and identified themes related to gender, food security, and nutrition using a thematic network approach (36). The codes were then reviewed to determine themes and sub-themes. The findings highlight the gender roles that men and women play in activities related to children’s nutrition.

The data were subjected to multiple readings by three authors. In these readings, particular attention was paid to the different roles men and women played in meeting the household’s food, care, and health needs in ensuring child nutrition. Again, this was an iterative process, which sometimes led to revising categorisation of themes and sub-themes. Throughout the process, these analyses were discussed and confirmed with the other authors, particularly the authors who conducted the fieldwork. The manuscript was also shared with CARE Malawi staff members who verified the accuracy of information related to the organisation and the related experiences of gender roles, food security, and nutrition in Malawi based on CARE’s ongoing work in the communities.

## Results

Table 2 provides a summary of participant demographic information highlighting the age range of the participants, educational attainment and number of children.

**Table 2:** Participant information

Participant group	Women			Mothers-in-law			Men		
**Age**	Lowest	Highest	Average	Lowest	Highest	Average	Lowest	Highest	Average
	18	39	28	40	75	56	21	40	34
**Educational attainment**	0	Secondary school completed	4 years of primary school	*Unknown*	*Unknown*	3 years of primary school	*Unknown*	*Unknown*	Secondary school completed
**Number of children**	1	6	3	*Unknown*			1	6	3

Generally, men and women play different roles in child nutrition. Table 3 lists activities that are performed by men and women. Both men and women shared their perceptions of who was responsible for each listed activity. Five main themes were identified. The first was productive work, which was defined as activities men and women participated in to obtain food. The second was reproductive work, which included housework and care work. Leisure, gender barriers to nutrition and undoing gender roles were the three additional themes that were identified.

**Table 3:** Summary of themes and sub-themes

Themes	Productive work: Activities to obtain food	Housework and care work	Leisure	Gender barriers to food security and nutrition	Undoing gender roles
**Sub-themes**	Business Sourcing food Farming	Childcare Cooking Drawing water Cleaning and other housework Household repairs and construction work		Women’s time constraints Barriers to men’s participation in “women’s work” Socialisation	*No sub-themes identified*

Eleven activities (sub-themes) were identified under the first three main themes. These activities are listed in the second column of Table 3. Men’s roles were mainly associated with four activities, including business, household labour (physical), leisure, and sourcing food. Women’s roles, on the other hand, were associated with nine activities. For some men, their participation in other activities was constrained by social norms with which they were raised. Men’s participation in these activities was exacerbated by barriers they faced when performing work typically associated with women.

### Productive work: activities to obtain food

Men and women were involved in three main activities to obtain food, including business, sourcing food and farming.

#### Business

More men than women mentioned involvement in business activities. Some men said that they had their own businesses such as tin pail making. These tin pails were used for drawing water. Others sold goods and were involved in carpentry. These activities generated income which could be used for food purchases. One woman in a FGD explained that the pail making business, which mainly involved men, was a source of income for food purchases. She said *“From this pail, you are able to find money for purchasing food.”* – P2, female.

Men who owned businesses invested much of their time and effort in business-related activities; while women’s role in business was only mentioned in one FGD, even though women felt that business was an opportunity to generate income for food, especially when food was scarce. One woman in a FGD in Kasakula said, *“When the woman sees that things are really hard in the home, she tries to find some money so that she can start a business.”* – P1, female.

#### Sourcing food

Sourcing food was mainly considered to be men’s responsibility. While many of the income-generating activities were to source food for the household, sourcing food is highlighted as a separate theme because participants emphasised the primary role of men in this specific activity. Much of men’s time was spent sourcing food by participating in *ganyu labour (*ganyu labour is a form of informal work, usually agricultural, that is compensated with money or food), farming, or hunting for mice. While both men and women expressed this sentiment, more men felt that sourcing food was their responsibility. Men were expected to use their income to buy food. In one FGD with men, participants were asked whose responsibility it was to buy relish and other food items. There was a general consensus that this was a man’s responsibility. One man, for example, said, *“But largely it is the man who has that responsibility.”* – P1, male.

Some of the items that participants mentioned that men bought included fish, meat, vegetables, maize, and sugar. For example, one woman in a focus group discussion in Kayembe said, *“The man sources relish and brings it like small fish, maybe meat.”* – P8, female.

Another man in a FGD in Dzoole said,*“The role of the man in the home is that he should source food in the home so that it is available, there should be good nutrition.”* – P3, male.

It was only in two cases where women mentioned that they were solely responsible for sourcing food. In these cases, both women were widowed. Some women felt that it was the responsibility of both men and women to source food. One woman from Kayembe said, *“In terms of sourcing food for the home, we source together. Whether it’s ganyu, then it means we do the ganyu together with the man.”* – P1, female.

A religious leader in Kalumo in an individual interview shared, *“Eee, in the home, if there is no food, then it means it’s both of them (man and woman) who take responsibility for doing ganyu, both men and women indeed. Where there is lack of food, then the man wakes up early to look (for food). The woman too wakes up early to look (for food). It is the job of everyone to source food, not just the man.”* **–** Individual interview, Male.

Men and women identified that they could source food by farming, finding ganyu labour or participating in business. One woman in a FGD in Kalumo said, *“For me, if I have gone out to ask for ganyu because we don’t have flour but then he also went elsewhere to look, but he didn’t find it. I tell him that I have found it and I show him the basin of flour. Then I ask him to go and help me. Then where I have found, we take our hoes and go and work together. We do work together.”* – P3, female.

#### Farming

Both men and women shared that they had a role to play in farming. Although more women than men expressed this joint role, both described situations where they participated in some form of farm work. One woman from Kalumo said, *“It’s the responsibility of both of us in the home. The one who starts off first in the morning is the man. I stay behind so that I can bathe the children, they should go to school. When I am finished is when I go to the garden.”* – P5, female.

A man from Kayembe shared that, *“In addition, we can say it’s both the man and the woman. Since you mentioned about farming, both are involved with farming work, the man does not do it alone.”* – P2, male.

In one FGD in Kasakula, the women joked that the rainy and ploughing seasons were a blissful time for marriage because they worked together with the men. However, when ploughing was completed, men were not as helpful. In some cases, men would leave their wives and marry another woman. One woman said, *“It really happens indeed. When you are finished ploughing, that’s it. You harvest, that’s the end of the marriage. This is what she means when she says the wife of the rainy season. The marriage is no longer there, no.”* – P3, female.

While farming was generally considered a shared responsibility, some women felt they were mainly responsible for farming. One woman from Kalumo said, *“For us women to farm on our own, it’s because of the laziness of the men. They refuse to go to the garden. That is why you find that we farm on our own. Yes, they are lazy.”* P2, female.

Alcohol presented a significant barrier to men’s participation in farming activities. One woman from Kasakula shared*, “When they are drunk? Then you will go to the garden while he (she used the term “it“; icho) is just there sleeping.”* – P7, female.

Another woman in the same FGD said*, “But also, when he is extremely drunk, in the morning, he wakes up with no strength at all. So, you just pick up your hoe, and you go to the garden, there you plough.”* – P5, female.

### Housework

Housework entailed five activities, including childcare, cooking, household labour, drawing water, and cleaning.

#### Childcare

Although it was reported that both men and women participated in childcare, this was mainly considered women’s responsibility. No men reported solely being responsible for this activity. Childcare, as women’s responsibility, was expressed mainly by men. Activities related to childcare included feeding (including breastfeeding) and bathing children, washing diapers, preparing food for children, washing baby clothes, and putting children to bed. One man from a FGD in Kalumo said,

*“It is different because when it comes to the child, the main person is the woman because if the child is breastfeeding, if you take him, then it means they will cry and will not stop soon. But when the child is with the mother, she will just start breastfeeding him. So, it means the main work is done by the mother.”* – P3, male.

Both men and women felt that childcare was a woman’s responsibility because women spent more time with children. When asked why only the mother could feed the child, a man from an FGD in Kalumo said, *“It is often the mother who stays with the child. As a man, you are out, but the child stays with the mother.”* – P4, male.

A mother-in-law in Kasakula shared that, *“ … a man does not walk with a child but a woman. Children are for a woman because a man does not go about with children. Breastfeeding, I am the one who breastfed. So, the one who goes about with children will pick up the hoe and go and do ganyu because she is the one who knows about the needs of a child.”* – P1, female.

Both men and women mentioned that men also play a role in childcare. Both men and women felt that men do and are capable of being involved in childcare. An equal number of men and women shared this sentiment. In one FGD with men in Kayembe, men were asked whose responsibility it was to care for infants. A few respondents felt that it was both men and women’s responsibility. Childcare activities that both men and women participated in included looking after the baby, cooking food for children, and bathing children. One woman in Kayembe said, *“Men who are understanding do play a role.” –* P4, female.

In some cases, men shared that they only became involved in childcare when the mother was busy. One man in Kayemba stated, *“We help with the baby if the mother is too busy.”* – P5, male.

One man from Kayembe shared that because he had provided financial resources, he had contributed towards childcare. He said, *“That is for both because if I go out and bring soap and my wife uses the soap to wash the children’s clothes, it means both of us have played a role.”* – P1, male.

#### Cooking

Similar to childcare, more men than women expressed that cooking was women’s responsibility. One mother-in-law in Kasakula said, *“The women are the ones who cook. They cook on their own even if they are tired. Here, it is the women who cook. Maybe in our friends’ areas, men are able to cook. But here in our area, women are the ones who cook.”* – P2, female.

More women than men expressed that cooking was sometimes a shared responsibility. One woman from Kayembe said, *“For me, I don’t experience abuse in my marriage. He helps me … cooking, feeding the children, chopping firewood.”* – P2, female.

One man from Kayembe shared that, *“But for me, I am able to help in cooking. At times I can tell my wife that today you can rest, I will do this one.”* – P4, male.

The other men laughed when he shared this comment. Most men felt that cooking was still predominantly women’s responsibility. However, there were certain circumstances under which men also cooked. These circumstances included when a woman was busy, ill, pregnant, or away from home. One man from Dzoole said, *“There are some chores that have stayed with women. Like here, for them to find you cooking, then it means the woman is sick, or she is away from the home.”* – P3, male.

A woman in Kasakula shared, *“It is us, women. We do not have the man cook, no. But when we are away, he cooks and also when we are sick is when he cooks.” –* P1, female.

#### Drawing water

Both men and women felt that drawing water was women’s responsibility. No men reported participation in collecting water. Women shared that water could be collected up to three times a day. This water was used for cooking, washing clothes, bathing, and washing dishes. One woman said, *“Maybe the water she drew in the morning is finished, she will go again to draw more water from the well.”* – P1, female.

Sometimes children helped women to collect water, which eased the workload of women. Even pregnant women were expected to collect water. In some cases, mothers-in-law helped when a woman was further along in her pregnancy. One woman in Kasakula shared*, “Women who are expecting fetch water a lot. If there are children, they help her. But for those who do not have children, the mother-in-law can also help when they see that the woman is very tired (with pregnancy).”* – P1, female.

#### Cleaning and other housework

More men than women felt that housework was women’s responsibility. Housework included: sweeping, washing dishes, collecting firewood, going to the maize mill, and washing clothes. One man in Kayembe said, *“She must clean the house when she wakes up, clean the plates, prepare porridge for the baby and for everyone then thereafter go farming.”* – P2, male.

A woman in Kayembe said, *“In the afternoon, the man goes to chat. Then I continue with the work. I should wash the dishes, put them in their place where they go. If there is something else that needs to be done, I do that, whether it’s washing, I should wash if that day things were not washed. I should also sweep the house after we have eaten nsima.*^4^*”* – P1, female.

Some men and women shared that housework is the responsibility of both sexes. Again, there were certain circumstances under which men participated in housework. One woman from Kayembe said, *“That is how I see it. When I am expecting, he helps me cook, chop firewood, even going to the mill.”* – P2, female.

#### Household labour

There are certain activities related to the physical maintenance of the home that were considered men’s responsibility. These activities were mentioned in four FGDs. Both men and women felt that women could not perform these specific activities because they required physical strength. One woman said that men were responsible for, *“Constructing houses. We women cannot manage.”* – P2, female.

Another man in a FGD in Kayembe said, *“The tasks must be different since we men do some things that women cannot do, and the women have tasks too that we can’t do. If we went to farm and come back, the wife will occupy herself with cooking while will we be busy constructing a tobacco shed. You can’t ask the wife to construct a tobacco shed which involves digging and climbing on a roof. So, our tasks are indeed different.”* – P1, male.

### Leisure

Men spent more time than women on leisure activities, especially when there was not much farm work to be completed. Men’s leisure time activities were identified mostly by men themselves however, both women and men noted that men’s leisure activities included chatting, playing bawo,^5^ drinking alcohol, playing soccer, watching sports, and resting. One man in a FGD in Kayembe said, *“Because we are not usually home, we are always up and about, like we said before that we go drinking, interacting with friends, playing soccer, meanwhile, the children are with their mothers.”* – P1, male.

Women were generally unable to enjoy the same amount of leisure time because of domestic responsibilities and socially-prescribed gender role expectations. One woman in Kayembe said, *“Men like going to chat more than women because you cannot go out the whole day and then the next day, the whole day, no. But for them, there is the opportunity to go to bawo. You continue your work in the home, you cannot leave. But the man goes to bawo.”* – P1, female.

Women had far less leisure time than men. Women’s leisure was only mentioned by a few men. Women’s leisure time included chatting with friends, going to church groups or choir, and reading the Bible. One woman from Kayembe said, *“When we eat in the afternoon, we have to chat. If you go to church groups, you need to go. If there is choir, you go and return and then come and take care of your home.”* – P3, female.

Table 4 summarises the gender distribution of work, how it relates to components of the UNICEF framework and the frequency with which the activities are performed. The summary illustrates that women perform more activities related to securing children’s nutrition. While men participate occasionally, activities related to childcare and healthy environment, in particular, are reserved for women. These activities are performed daily. In rural Malawi, limited infrastructure means that more time and energy is spent on activities such as collecting water and firewood.

**Table 4:** Gender distribution of work and frequency

UNICEF framework component	Type of work	Men	Women
Access to food	Business	Daily	
	Sourcing food	Daily	Daily
	Farming	Seasonal	Seasonal
Care for women and childcare	Childcare	Occasionally	Daily
	Cooking	Occasionally	Daily
Healthy environment	Drawing water		Daily
	Cleaning and other housework	Occasionally	Daily
Not applicable	Household repairs and construction work	Occasionally	

### Gendered barriers to child nutrition

The gendered roles men and women play in food, care, and health often constrained their ability to meet children’s nutrition needs. These included women facing time constraints and barriers to men performing work that is typically associated with women.

#### Time constraints and barriers to men performing women’s work

Women’s multiple roles often meant that they made trade-offs between performing their household responsibilities or sourcing food. As previously noted, collecting water was one of the tasks that women felt was particularly time-consuming. Women also shared that they could not freely pursue ganyu labour because of household responsibilities. One woman from Dzoole said, *“The difference is that we are busy, so the ganyu that we do is limited. We have to first do household chores, compared to men (who don’t).”* – P8, female.

Societal expectations of men often prevented them from supporting women in reducing their work burdens. Some men experienced shaming if they performed work that was associated with women. This fear of backlash prevented men from performing this work when women were home. A man from Dzoole explained that you only help with household chores when the woman is away*, “But if she is there, then people will disrespect you that your wife has fed you medicine. In refusing the saying that they have fed you medicine, this is why women are suffering with these chores.”* – P3, male.

These gender roles appeared to be learned through a process of socialisation from childhood and a lack of exposure to role models who did not follow traditional gender norms. One man in a FGD in Kalumo said, *“The difference between this work … like us, we also observed from our parents because like in the area of cooking like this then it means our parents, they have never really been involved with this issue of gender so that some things we take it from our parents, differentiating work.”* – P5, male.

Keeping things the same, as they were in the past, challenges changing gender norms. As another man from Kayembe said, *“This is how it has been from the past, that a woman must cook.”* – P1, male.

Mothers-in-law from Kasakula shared that their children behaved a certain way because they learned these behaviours from their parents. One woman said, *“A chick imitates what the mother hen does. She is doing it, and the child is there. You cannot say it will not see. It will see.”* – P1, female.

### Undoing gender roles

The results of the first three themes suggest that some activities, although considered women’s responsibility, were performed to some extent by both sexes. There was an indication that men and women were subverting some of the socially-prescribed traditional gender roles. When asked how this change came about, one woman from Dzoole said, *“It started some years back when the issue of gender just came. They [men] actually changed.”* – P7, female.

Women suggested that both men and women had an equal role to play in the home. One woman from Kayembe said, *“Yes, it was different in the past. In the past, the one who was seen to be at the forefront was the man. But today, no. Everyone has their own role. The man needs to participate in whatever way will be helpful. The woman needs to participate in whatever way will be helpful.”* – P3, female.

Another woman from Kayembe said, *“In the past, it was different from nowadays. In the past, they said the man is the head of the family. But today, “gender“ has come. Everyone has the right to play a role.”* – P1, female.

One woman from Kayembe mentioned that she is part of an organisation that offers education on gender. They teach the communities that there is no difference between a man and a woman and that women can also perform men’s tasks. She said, ***“****Things indeed, have changed. They are different from what it was like. Even in the home, you find that when the man is not there, and the roof is leaking, you just say, there is just need for some paper, bring a stick. And you climb up indeed, you take a paper, and you place it there. Then it means everything is well there.”* – P4, female.

A man in a FGD in Kalumo said, *“It’s important that when it comes to the role of cooking, we all need to play a role because there are some of us men, we just pressure women that you should go cook when perhaps the woman is also doing other work. Yet you have the opportunity, you have time that you can also take over from the woman, it doesn’t mean that you will not have intelligence, no. But household chores are about helping one another, both of you want to work towards good nutrition in the home.”* – P5, male.

## Discussion

Literature highlights that women play a greater role in children’s nutrition [34, 37–39]. However, little is understood about women’s role in relation to men’s role regarding child nutrition. Our study found that men’s and women’s roles in child nutrition are typically gendered, with more women participating in food, care, and health-related activities. The increased work burden women carry in meeting children’s nutrition needs threatens positive child nutrition outcomes as they often have to navigate generating income versus food preparation. Our findings suggest that men could also play an essential role in children’s nutrition. While this role can reduce women’s work burden, men’s shared responsibility in child nutrition is insufficient for overcoming the structural inequalities that undermine gender equality.

Concerning food, both men and women participated in several activities to obtain food for the household. Although more men reported owning businesses than women, there was an indication that some women were involved in business activities to generate income. Sourcing food was primarily seen as men’s responsibility. However, women frequently participated in this activity too. Men also typically had more time to generate income, given women’s large household burden. As such, there was an expectation that men would, or rather should, be responsible for purchasing food. This finding is consistent with literature that suggests that Malawian men are typically responsible for sourcing food [29, 33]. The food items that men were said to buy increased the diversity of food consumed in the household. This food can significantly improve household nutrition.

Farming was generally perceived as both men’s and women’s responsibility. Marriage patterns in rural Malawi tend to be tenuous and are informed by the harvest. Some women shared that they carried the primary responsibility for farming. Alcohol and laziness were considered two of the main reasons for men’s lack of participation in farming. Given the multiple responsibilities women carry, they rarely had time for leisure. On the other hand, men spent a significant amount of time on leisure, mainly when it was not farming season. Women expressed frustration with men who did not offer adequate contributions to farming. They also raised concern that some men did not provide sufficient support financially or with household responsibilities. This finding is consistent with Kerr et al. [40], who found that women were not only concerned about lack of adequate food, they were also concerned with their partner’s behaviours that worsened food insecurity.

Concerning care, men and women suggested that children needed to be with the mother because women breastfeed. Consequently, childcare was considered women’s responsibility because women spent more time with children. Men would only help with childcare under certain conditions. The same applied to cooking. Men would only help with cooking if women were busy, sick, or away from home. The analysis indicates that the mothers-in-law seem to hold and perpetuate expected gender roles. Women regarded a man who helped with childcare as understanding. Similarly, women felt that men who helped with cooking were not abusive, reinforcing the idea that these were still women’s responsibilities. This finding is consistent with Pierrioti et al. [41], who suggest that the notion that men were “helping” reinforces the belief that specific roles are reserved for women and others for men. While men sometimes take up work that is typically associated with women, gender equality can only be realised if men are willing to share household duties and relinquish some form of power and control over resources within the household.

The likelihood that women might carry out multiple activities simultaneously, especially childcare, is important to note. Women explained that they frequently went to ganyu labour with their children. When women described farming or business activities they participated in, it was unclear where the children were during this time. As Lentz et al.(2019) [19] suggest, the time spent on childcare was not necessarily captured because women were not always prompted to discuss these issues. However, women are likely performing childcare activities and farm work simultaneously, especially in cases where social support systems, such as grandmothers to look after children, are weak. However, even in cases where social support systems are available, challenges persist when a child is still breastfeeding. The fact that childcare is ignored reinforces the idea that women and men do not acknowledge the value of unpaid work, particularly care work, because it generates no income.

Drawing water, washing dishes and clothes, and cleaning the house are all activities that contribute to the household’s health environment. Women mainly take up these activities. When women and men were asked if men participated in certain activities that society typically considered women’s work, some women said that men had played a role by buying soap. This finding reinforces the notion that paid work is more valued. Even though the man is not actively involved in household work, he has contributed to household health because he has provided the financial resources needed to make this possible.

While some men participated in reproductive work, this was still not considered the norm. Men did not explicitly articulate that women’s workloads were more cumbersome than theirs, they explained that they could not participate in these activities because it was not the norm. Societal gender norms continue to perceive women’s heavy workloads as normal [18], failing to understand the underlying negative impacts these have on childcare and, ultimately, health and nutrition. Women’s work burden and men’s lack of participation sometimes resulted in women not preparing food or attending to other housework. Consequently, children frequently missed meals when women were not around. While some men were able to cook, most felt that this work was reserved for women. The extent to which men prepared food for children when women were not around could have been further investigated with additional probing. However, it was clear that traditional gender roles were no longer strictly adhered to in some cases.

**Fig. 1:**
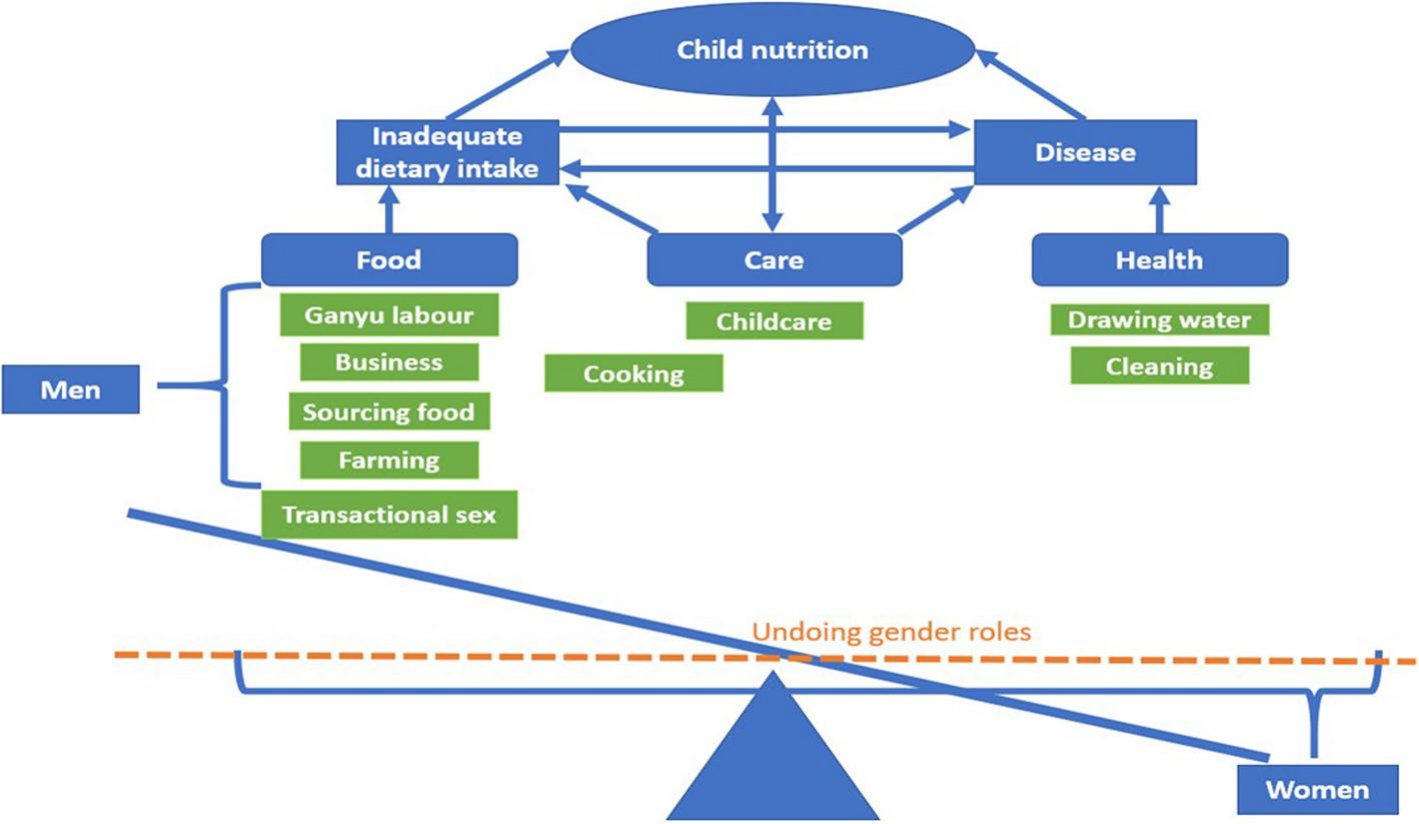
Women’s work burden in child nutrition

Men and women indicated that some men were involved in cooking, cleaning, housework, and childcare. These activities are contrary to the traditional gender roles described in the literature (12, 31). Gender advocacy has made advancements, particularly when these activities are embraced from within the community. However, this finding was specific to one community where programming enabled people to engage differently and to speak to issues of gender in a more informed way. This finding is not necessarily representative of the rest of the country. Other studies from Ntcheu and Northern Malawi, with similar programming, indicate some advances in undoing traditional gender roles [30, 42, 43]. In these cases, men recognised the need to support women with housework. The support they offered could alleviate women’s workload. This support can allow women more time to focus on children’s nutrition, but also offer men opportunities to become involved in supporting children’s nutrition. Drawing on our analysis, Fig. 1 illustrates how the imbalance in gender roles takes women further away from providing optimal nutrition for children and women. Levelling out these work burdens could increase men’s and women’s capacity to support child nutrition.

While women’s might become less constrained, and they may be able to pursue other income-generating activities, this may not necessarily improve their power in the household. A study by Handa et al. [44] indicates that when women’s income increases, they only have increased control over their income and not necessarily the household’s pooled income. Re-allocating or redistributing gender roles alone is not sufficient. As Ganle and Dery [45] point out, some women may not want men to be involved in women’s work as it may threaten their autonomy. Therefore, it is essential that communities, particularly women, are engaged in processes to define how they would like to see gender roles and norms evolve to transform social systems that underpin inequalities.

### Limitations

This study was only conducted in two communities in rural Central Malawi. While the findings can be applied to similar contexts, the purpose of the study was to gain an in-depth understanding of gender dynamics in these communities to support CARE in defining programme interventions. Quantitative data could have complemented the study, particularly in linking the roles that women played with child nutrition outcomes. The endline survey will include the collection of quantitative data to complement the qualitative aspects. The study could also have sampled more men to generate insights on how men’s involvement in unpaid care can improve children’s nutrition outcomes. Despite the limited number of men sampled, the themes included were representative of all three communities. The authors made explicit efforts to ensure that when a theme was coded, it was relevant to all three communities. The authors also drew on existing literature to corroborate and validate the findings from this study and to increase credibility.

Further studies could integrate quantitative data to understand the extent to which men participate in child nutrition. Such data could be aligned with nutrition outcomes to assess if men’s participation in child nutrition improves child nutrition outcomes. Further research is needed to explore how community-led efforts to redistribute gender roles can also encourage men to share decision-making power and control over resources.

## Conclusions

Traditional gender roles remain prevalent in rural Central Malawi, which impacts child nutrition. Women are primariy and simultaneously responsible for the food, care, and health of the household. Women’s heavy workloads prevent them from providing optimal care and nutrition for children. However, men do offer support when women are overburdened or away from home. The extent to which men offer this support needs to be quantitatively understood.

While efforts to redistribute gender roles appear to have had marginal success, the extent to which these efforts have successfully encouraged men to share power remains unclear. Men are taking on tasks that have typically been associated with women. The success of these interventions depends on women having more time to pursue income-generating activities, which may or may not increase their power within the household. Efforts to increase men’s participation in “women’s activities” need to be complemented with community-led processes that define how gender roles can change to improve gender power relations within the household and society at large.

## Data Availability

The data is not available to be shared publicly as it could compromise the privacy of research participants, but are available from Clement Bisai at CARE Malawi on reasonable request.

## Footnotes

1 Ganyu labour is a form of informal work, usually agricultural, that is compensated with money or food.

2 Mice are a good source of protein particularly in the rural areas. Men and boys frequently hunt for and trap mice for household food.

3 Relish is eaten together with the cornmeal staple nsima, usually in the form of vegetables or beans or a protein the family can afford.

4 Nsima is a thick paste made of maize or corn flour that is eaten as a staple throughout Malawi and other South East African countries. It is eaten along with vegetables, beans and if available, a protein such as meat or eggs.

5 Bawo is a traditional board game played in most of East Africa including Kenya, Tanzania, Comoros, Malawi, as well as some areas of DR Congo and Burundi.

## References

[1] FAO (2020). The state of Food Security and nutrition in the world 2020 - transforming food systems for affordable healthy diets.

[2] Blössner M, de Onis M (2005). Malnutrition: quantifying the health impact at national and local levels. WHO environmental burden of disease series, no. 12.

[3] Ltd DIPR (2021). 2021 global nutrition report: the state of global nutrition.

[4] UN (1976). UN Convention on economic social and cultural rights.

[5] Hoddinott J (2016). The economics of reducing malnutrition in sub-Saharan Africa. Global panel on agriculture and Food Systems for Nutrition Working Paper.

[6] AUC (2015). Africa Agenda 2063: First ten-year Implementation Plan 2013–2023.

[7] Kihiu EN, Amuakwa-Mensah F (2020). Agricultural market access and dietary diversity in Kenya: gender considerations towards improved household nutritional outcomes. Food Policy.

[8] Twynman J (2020). Gender equity considerations in Food environments of low and middle income countries.

[9] Bargain, O., P. Kwenda-Magejo, and M. Ntuli, Gender bias and the intrahousehold distribution of resources: evidence from African nuclear households in South Africa. WIDER working Paper, no. 2017/71, in Helsinki. 2017, United Nations University World Institute for Development Economics Research (UNU-WIDER).

[10] UNICEF, UNICEF’s approach to scaling up nutrition for mothers and children. 2015: New York.

[11] Security, C.o.W.F., Coming to terms with terminology. 2012: Rome.

[12] Cislaghi B, Heise L (2020). Gender norms and social norms: differences, similarities and why they matter in prevention science. Sociol Health Illn.

[13] Choudhary N, Parthasarathy D (2007). Gender, work and household Food Security. Econ Polit Wkly.

[14] Naz M, Khan IA, Shahbaz B (2014). Role of rural women in agriculture and household food security in Faisalabad district. Pakistani J Agri Sci.

[15] Rao, N., A. Pradhan, and D. Roy. Gender Justice and Food Security in India: A review. IFPRI Discussion Paper 01600, Washington DC. 2017: Washington, DC.

[16] Doss C (2018). Women in agriculture: four myths. Glob Food Sec.

[17] Doss, C., If women hold up half the sky, how much of the world’s food do they produce? ESA Working Paper No 11–04. 2011: Rome.

[18] Kerr RB (2005). Food Security in northern Malawi: gender, kinship relations and entitlements in historical context. J South Afr Stud.

[19] Lentz E (2019). The invisible hand that rocks the cradle: on the limits of time use surveys. Dev Chang.

[20] Geere J, Cortobius M (2017). Who carries the weight of water? Fetching water in rural and urban areas and the implications for water security.

[21] Shaw S. Women’s lives around the world: A global encyclopedia. 4 ed, ed. S. Shaw. 2018. Conecticut: Greenwood Publishing Group.

[22] UNICEF and WHO (2015). Progress on sanitation and drinking water – 2015 update and MDG assessment.

[23] Graham JP, Hirai M, Kim S-S (2016). An analysis of water collection labor among women and children in 24 sub-Saharan African countries. PLoS One.

[24] ICF, N.S.O.a.M., Malawi Demographic and Health Survey 2015–16. 2017, NSO and ICF Macro: Zomba and Maryland.

[25] Fleifel E, Martin J, Khalid A (2019). Gender specific vulnerabilities to water insecurity.

[26] Kayser GL (2019). Water, sanitation and hygiene: measuring gender equality and empowerment. Bull World Health Organ.

[27] Morrell R (2016). Fathers who care and those that don’t: men and childcare in South Africa. S Afr Rev Sociol.

[28] (UNGA), U.N.G.A., Transforming our world: The 2030 agenda for sustainable development. General Assembley 70 session. 2015, UNGA: New York.

[29] Manda-Taylor L (2017). Changing times? Gender roles and relationships in maternal, newborn and child health in Malawi. BMC Pregnancy Childbirth.

[30] Mkandawire E, Hendriks SL (2019). “The role of the man is to look for food”: lessons from men’s involvement in maternal and child health programmes in rural Central Malawi. PLoS One.

[31] Akanle O, Adesina JO, Ogbimi AO (2016). Men at work keep-off : male roles and household chores in Nigeria. Gender Behav.

[32] Ministry of Finance, E.P.a.D., Malawi Growth and Development Strategy (MGDS) II Review and Country Situation Analysis Report. 2016, Minitry of finance, Economic Planning and Development, Government of Malawi: Lilongwe.

[33] Mkandawire E, Hendriks SL (2018). A qualitative analysis of men’s involvement in maternal and child health as a policy intervention in rural Central Malawi. BMC Pregnancy Childbirth.

[34] Ragasa C, Aberman N-L, Alvarez Mingote C (2019). Does providing agricultural and nutrition information to both men and women improve household food security? Evidence from Malawi. Glob Food Security.

[35] Schipanski ME (2016). Realizing resilient Food systems. BioScience.

[36] Macro, N.S.O.a.I., Malawi Demographic and Health Survey. 2010, NSO and Macro: Zomba and Maryland.

[37] Abuya BA, Ciera J, Kimani-Murage E (2012). Effect of mother’s education on child’s nutritional status in the slums of Nairobi. BMC Pediatr.

[38] Aberman N-L, Roopnaraine T (2020). To sell or consume? Gendered household decision-making on crop production, consumption, and sale in Malawi. Food Security.

[39] Smith LC (2003). The importance of women’s status for child nutrition in developing countries.

[40] Kerr, R.B., E. Lupafya, and L. Shumba, Food Sovereignty, Gender and Nutrition: Perspectives from Malawi. Conference Paper #68. 2013, Program in Agrarian Studies, Yale University. Conference paper for discussion at: International Conference September 14–15, 2013.: New Haven.

[41] Pierotti RS, Lake M, Lewis C (2018). Equality on his terms: doing and undoing gender through Men’s Discussion groups. Gend Soc.

[42] Kerr RB (2016). Integrated agriculture programs to address malnutrition in northern Malawi. BMC Public Health.

[43] Mkandawire E, Hendriks SL (2018). A gender assessment of Malawi's National Nutrition Policy and Strategic Plan 2007–2012. Dev Policy Rev.

[44] Handa S (2009). Opening up Pandora’s box: the effect of gender targeting and conditionality on household spending behavior in Mexico’s Progresa program. World Dev.

[45] Ganle JK (2016). 'If I go with him, I can't talk with other women': understanding women's resistance to, and acceptance of, men's involvement in maternal and child healthcare in northern Ghana. Soc Sci Med.

